# AKR1C3 protects cardiomyocytes against hypoxia-induced cell apoptosis through the Nrf-2/NF-κB pathway: AKR1C3 protects against cardiomyocyte apoptosis

**DOI:** 10.3724/abbs.2024230

**Published:** 2025-03-06

**Authors:** Wenlu Zhang, Wei Tian, Xin Xia, Hua Tian, Ting Sun

**Affiliations:** 1 Department of Cardiology Shanghai Ninth People’s Hospital Shanghai Jiao Tong University School of Medicine Shanghai 200025 China; 2State Key Laboratory of Systems Medicine for Cancer Shanghai Cancer Institute Renji Hospital Shanghai Jiao Tong University School of Medicine Shanghai 20032 China; 3 Department of Pathology the Affiliated Hospital of Youjiang Medical University for Nationalities Baise 533000 China; 4 Key Laboratory of Molecular Pathology in Tumors of Guangxi Higher Education Institutions Baise 533000 China

**Keywords:** AKR1C3, hypoxia, cardiomyocyte apoptosis, Nrf-2, NF-κB

## Abstract

Hypoxia-induced apoptosis plays a critical role in the progression of various cardiac diseases, such as heart failure and acute myocardial infarction (AMI). Aldosterone reductase 1C3 (AKR1C3), a member of the aldo-keto reductase superfamily, participates in the metabolism of steroid hormones and redox reactions *in vivo*. Imbalances in prostaglandin levels have been linked to coronary events. However, the function and molecular mechanism by which AKR1C3 influences AMI are not yet fully understood. This study aims to investigate the role of AKR1C3 in hypoxia-induced myocardial cell damage and elucidate its mechanism. Our findings reveal that a hypoxic microenvironment triggers cardiomyocyte apoptosis and elevates AKR1C3 expression in H9C2 and AC16 cells, as well as in cardiac tissue from rats and mice with AMI. The overexpression of AKR1C3 promotes cardiomyocyte proliferation and cell vitality, whereas the silencing of *AKR1C3* exerts the opposite effects *in vitro*. AKR1C3 protects cardiomyocytes against hypoxia-induced cell apoptosis by reducing ROS levels, preventing mitochondrial damage, and maintaining the oxygen consumption rate (OCR) and ATP production; conversely, *AKR1C3* knockdown leads to adverse outcomes. Moreover, the application of a ROS inhibitor (MitoQ10) mitigates the increase in mitochondrial ROS in cardiomyocytes induced by *AKR1C3* knockdown under hypoxic conditions. Mechanically, AKR1C3 increases Nrf-2 expression through the ubiquitin-proteasome pathway in cardiomyocytes and subsequently inhibits the NF-κB signaling pathway, thereby inhibiting Bax/caspase-3 signaling. Collectively, these results suggest that AKR1C3 prevents hypoxia-induced cardiomyocyte injury by modulating the Nrf-2/NF-κB axis, suggesting new insights into the mechanisms underlying myocardial protection.

## Introduction

Heart failure is the main cause of disability and death after myocardial infarction [1]. During the early stages of acute myocardial infarction (AMI), apoptosis is recognized as a key contributor to myocardial cell loss after AMI, serving as a pivotal factor in the initiation of ventricular remodeling and the progression of heart failure [2]. Our previous study demonstrated that integrin β3 exerts a protective effect on cardiomyocytes during hypoxia-induced apoptosis [3]. The heart is an organ with a high demand for energy, and mitochondria serve as the principal source of adenosine triphosphate (ATP) and reactive oxygen species (ROS) in cardiomyocytes. Disruption of oxidative phosphorylation and excessive production of ROS increase cardiomyocyte apoptosis [4]. Therefore, exploring the mechanism of hypoxia-induced cardiomyocyte apoptosis is crucial.

Mitochondrial ROS has always been a therapeutic target for preventing hypoxic injury. Under physiological conditions, ROS serves as the second messenger in signal transduction pathways, participating in the regulation of oxidative balance and various biological activities. However, excessive ROS production can lead to oxidative stress and damage [5]. Pathological stimuli, such as hypoxia, can trigger a “burst” release of mitochondrial ROS, disrupting the endogenous antioxidant balance, ultimately resulting in mitochondrial depolarization and mitochondrial outer membrane permeabilization (MOMP) and ultimately leading to apoptosis [6]. Aldosterone reductase 1C3 (AKR1C3) belongs to the aldosterone reductase (AKR) superfamily and has a strong affinity for NADPH, primarily facilitating redox reactions [7]. AKR1C3 is predominantly expressed in endocrine organs such as the prostate, adrenal gland, mammary gland, and uterus. AKR1C3 also participates in the *de novo* synthesis of steroids in the adrenal gland and tumors [8]. Many studies have revealed the role of AKR1C3 in tumor progression [ 9– 12]. In addition, AKR1C3 was found to induce chemoresistance by regulating redox balance and ROS production in malignant tumor cells [13]. AKR1C3 functions as a prostaglandin (PG)F _2_ synthase, modulating vasodilation and vasoconstriction [14]. Imbalances in prostaglandins have been associated with coronary events [ 15, 16]. Despite previous studies reporting no significant difference in AKR1C3 expression between AMI patients and controls on the basis of the GSE48060 dataset, these findings may be limited by small sample sizes [17]. Thus, further investigations are needed to understand the relationship between AKR1C3 and AMI comprehensively.

In the present study, we investigated the precise mechanisms by which AKR1C3 influences cardiomyocyte proliferation and protects against hypoxia-induced cardiomyocyte apoptosis, thereby contributing to a broader understanding of myocardial protection strategies.

## Materials and Methods

### Cell lines, cell culture and cell treatment

The rat embryonic cardiomyocyte cell line H9C2 was purchased from the Cell Bank of the Institute of Biochemistry and Cell Biology of the Chinese Academy of Sciences (Shanghai, China). The human cardiac cell line AC16 was purchased from American Type Culture Collection (ATCC; Rockville, USA). These cell lines were continuously cultured in Dulbecco’s modified Eagle’s medium (DMEM) supplemented with 12.5% fetal bovine serum (FBS) at 37°C and 5% CO _2_. To induce hypoxic injury in H9C2 and AC16 cardiomyocytes, H9C2 and AC16 cardiomyocytes were plated in serum-free medium and cultured in a hypoxic incubator (Whitley H35 hypoxystation; Don Whitley Scientific, West Yorkshire, UK) with 95% N _2_, 5% CO _2_, and 1% O _2_ for 12 h at 37°C or treated with CoCl _2_ (600 μM) for 12 h. Cardiomyocytes in the normoxic control group were cultured in a normoxic incubator with 95% air and 5% CO _2_ at 37°C.

### Plasmids, lentivirus production and lentivirus infection

To generate stable AKR1C3-overexpressing cell lines, the AKR1C3 open reading frame (ORF) sequence was PCR amplified via specific primers (forward: 5′-ATGGATTCCAAACACCAGTGT-3′, and reverse: 5′-TTAATATTCATCTGAATATGG-3′) and cloned and inserted into the lentiviral expression vector pWPXL (Addgene, Watertown, USA). Short hairpin RNAs (shRNAs) targeting AKR1C3 and a negative control (NC) shRNA were supplied by GeneChem (Shanghai, China). The target sequence of AKR1C3-e was 5′-GGTGAGGAACTTTCACCAACA-3′. The target sequence of AKR1C3-f was 5′-GGTAGAATGTCATCCGTATTT-3′. The target sequence of NC was 5′-TTCTCCGAACGTGTCACGTT-3′. Lentiviruses were generated by transfecting HEK-293T cells in 60-mm cell culture dishes with the lentiviral vector, pMD2 (Addgene) or psPAX2 (Addgene) using Lipofectamine 2000 transfection reagent (Invitrogen, Carlsbad, USA). The supernatants containing the AKR1C3-overexpressing and *AKR1C3*-knockdown lentiviruses were then harvested after 48 h and filtered through a sterile 0.45-μm syringe. H9C2 and AC16 cells were infected with 1 × 10 ^6^ recombinant lentivirus-transducing units in the presence of 6 μg/mL polybrene (Sigma, St Louis, USA).

### Quantitative real-time PCR (qRT-PCR)

Total RNA was extracted using Trizol total RNA isolation reagent (Invitrogen) according to the manufacturer’s instructions. Reverse transcription and qRT-PCR were performed as previously described [11] using an ABI Prism 7500 System (Applied Biosystems, Foster City, USA) with SYBR® Premix Ex Taq (Takara, Dalian, China). The sequences of primers used were as follows: *AKR1C3* (human)-F: 5′-ACGATGGGTGGACCCGAA-3′, *AKR1C3* (human)-R: 5′-CGTTCTGTCTGATGCGCTGC-3′; *Nrf-2* (human)-F: 5′-CAACTACTCCCAGGTTGCCC-3′, *Nrf-2* (human)-R: 5′-CAACAGGGGCTACCTGAGC-3′; and *β-actin* (human)-F: 5′-AGGCATCCTGACCCTGAAGTAC-3′, *β-actin* (human)-R: 5′-GAGGCATACAGGGACAACACAG-3′. The results for the expression of *AKR1C3* are presented relative to the expression of the *β-actin* gene according to the delta-delta Ct method.

### Western blot analysis

Total proteins extracted from cells were separated by 8%–12% SDS-PAGE and transferred to polyvinylidene difluoride membranes (Bio-Rad, Hercules, USA). After being blocked with 5% nonfat milk, the membranes were incubated with specific antibodies at 4°C overnight and probed with HRP-conjugated secondary antibodies (Proteintech, Wuhan, China). The primary antibodies used were against AKR1C3 (1:500; Abcam, Cambridge, UK), phospho-p65 (1:500; Cell Signaling Technology, Danvers, USA), p65 (1:1000; Cell Signaling Technology), phospho-IκBα (1:500; Cell Signaling Technology), IκBα (1:500; Cell Signaling Technology), Nrf2 (1:500; Cell Signaling Technology), Bcl-2 (1:500; Proteintech), Bax (1:500; Proteintech), cleaved caspase 3 (1:250; Cell Signaling Technology) and β-actin (1:10,000; Sigma). Enhanced chemiluminescence reagent (Pierce, Rockford, USA) was used for visualization, and detection was performed with a Bio-Rad instrument. β-Actin served as a loading control.

### Immunoprecipitation

Hypoxia-treated AC16 cells were harvested in RIPA (containing protease inhibitor) lysis buffer for 60 min at 4°C and centrifuged at 12,000 *g* for 10 min. A small amount of supernatant was used for western blot analysis, and the remaining lysate was supplemented with antibodies against Nrf-2 and protein A/G agarose beads overnight at 4°C while rotating. After the immunoprecipitation reaction, the protein A/G beads were centrifuged at 3000 *g* for 5 min at 4°C, the supernatant was carefully removed, the protein A/G beads were washed with lysis buffer 3 times, and the complexes were subjected to western blot analysis.

### Immunohistochemistry (IHC)

Immunohistochemical staining was performed as described previously [18]. The sections were deparaffinized with xylene and rehydrated in alcohol. Endogenous peroxidase was blocked with 3% hydrogen peroxide and then incubated with the indicated antibodies (anti-AKR1C3; Sigma) overnight at 4°C. A HRP-conjugated secondary antibody was used, and then a DAB kit (Thermo Fisher Scientific, Waltham, USA) was used for signal detection.

### Cell proliferation, cell viability and colony formation assays

Cell proliferation was measured by EdU assay (KTA2031; Abbkine, Wuhan, China). Briefly, the treated H9C2 cells were incubated with 10 μM EdU for 3 h. Then, cells were fixed, permeabilized, and stained with Click-iT reaction in the dark at 37°C for 30 min. The nuclei were stained with Hoechst and visualized under a fluorescence microscope. Cell viability was assessed by Cell Counting Kit-8 (CCK-8; Bimake, Houston, USA) according to the manufacturer’s instructions. The cells were seeded in 96-well plates (1000 cells per well for the cell proliferation test; 3000 cells per well for the cell viability test) and treated with normoxia, hypoxia or CoCl _2_ (600 μM) for 12 h. The culture medium was replaced by 100 μL of fresh medium containing 10 μL of the CCK8 solution for each well. The plate was returned to the cell culture incubator for 2 h. Next, the absorbance at 450 nm was measured via a microplate reader (Thermo Fisher Scientific). For the colony formation assays, 5 × 10 ^3^ cells were plated in each well of a 6-well plate and incubated at 37°C for 2 weeks. Colonies were fixed with 4% phosphate-buffered formalin (pH 7.4) and stained with Giemsa for 15 min, and counted. Each experiment was performed in triplicate.

### Immunofluorescent confocal imaging

The cells were plated on coverslips and fixed with 4% paraformaldehyde for 20 min at room temperature. Then, the cells were permeabilized with 0.5% Triton X-100 for 20 min and blocked with 5% BSA solution for 1 h. The slides were incubated with primary antibodies in a blocking solution overnight at 4°C in a humidified chamber. The glass slides were subsequently washed three times in PBS and incubated with Alexa Fluor 488-conjugated secondary antibodies (Thermo Fisher Scientific) and 4′,6-diamidino-2-phenylindole (DAPI) in a blocking solution for 30 min at 37°C in a humidified chamber. Images were acquired with a Leica TCS SP8 confocal system (Leica, Wetzlar, Germany).

### Flow cytometry analysis

The percentage of apoptotic cells was determined using the Annexin V-PE/7-AAD Apoptosis Detection kit (BD Biosciences, San Jose, USA). After the indicated treatments, AC16 and H9C2 cells were collected using trypsin and gently washed with ice-cold phosphate-buffered saline before being suspended in 100 μL of binding buffer containing 5 μL of PE-conjugated Annexin-V and 5 μL of 7-AAD. After incubation in the dark on ice for 15 min, apoptotic cells were analyzed by flow cytometry. The same procedure was repeated for subsequent analyses.

### Oxidative stress detection and measurement of mitochondrial reactive oxygen species (ROS)

ROS production was evaluated by DCFH-DA staining. Briefly, AC16 cells were treated and then stained with DCFH-DA (Invitrogen) in the dark at 37°C for 30 min. The cells were subsequently analyzed by flow cytometry.

To detect superoxide in the mitochondria of live cells, MitoSOX Red reagent (Invitrogen) was used. The cells were incubated with MitoSOX Red working solution in the dark at 37°C and 5% CO _2_ for 30 min and then analyzed by flow cytometry.

### Detection of the mitochondrial membrane potential (MMP)

JC-1 was used to monitor the MMP. An enhanced mitochondrial membrane potential assay kit (Beyotime, Shanghai, China) was used to detect changes in the MMP in H9C2 and AC16 cells. In brief, 1 mL of JC-1 working solution was added to the cells in 6-well plates and incubated in a CO _2_ incubator at 37°C for 20 min. After incubation, the supernatant was aspirated, and the cells were washed twice with staining buffer. Two milliliters of cell culture medium was added before observation under a fluorescence microscope (Leica). The red/green fluorescence ratio of the dye in the mitochondria can be considered a direct assessment of the state of the MMP.

### Measurement of the mitochondrial oxygen consumption rate (OCR)

AC16 cells (5 × 10 ^3^ cells per well) were seeded on an XF96 cell culture plate (Agilent, Santa Clara, USA) and cultured for 24 h. The mitochondrial OCR was evaluated using a MitoStress Test kit (Cat. No. 103015-100; Agilent) [19]. Oligomycin, FCCP and a mixture of rotenone and antimycin A were added according to the manufacturer’s instructions and protocols. The calibration plate and the probe plate were subsequently placed into Agilent’s Seahorse Bioscience XF96 Extracellular Flux Analyzer (Agilent). The OCR was calculated via Wave software (Agilent).

### Statistical analysis

All the data are presented as the mean ± SD. Unpaired Student’s *t* tests were used to compare the means of the two groups. One-way analysis of variance was used for comparisons among the different groups. Statistical analyses were performed via SPSS 19.0 software (SPSS Inc., Chicago, USA). *P*  < 0.05 was considered statistically significant.

## Results

### Hypoxia-induced cardiomyocyte apoptosis is associated with increased AKR1C3 expression

**Figure 1:**
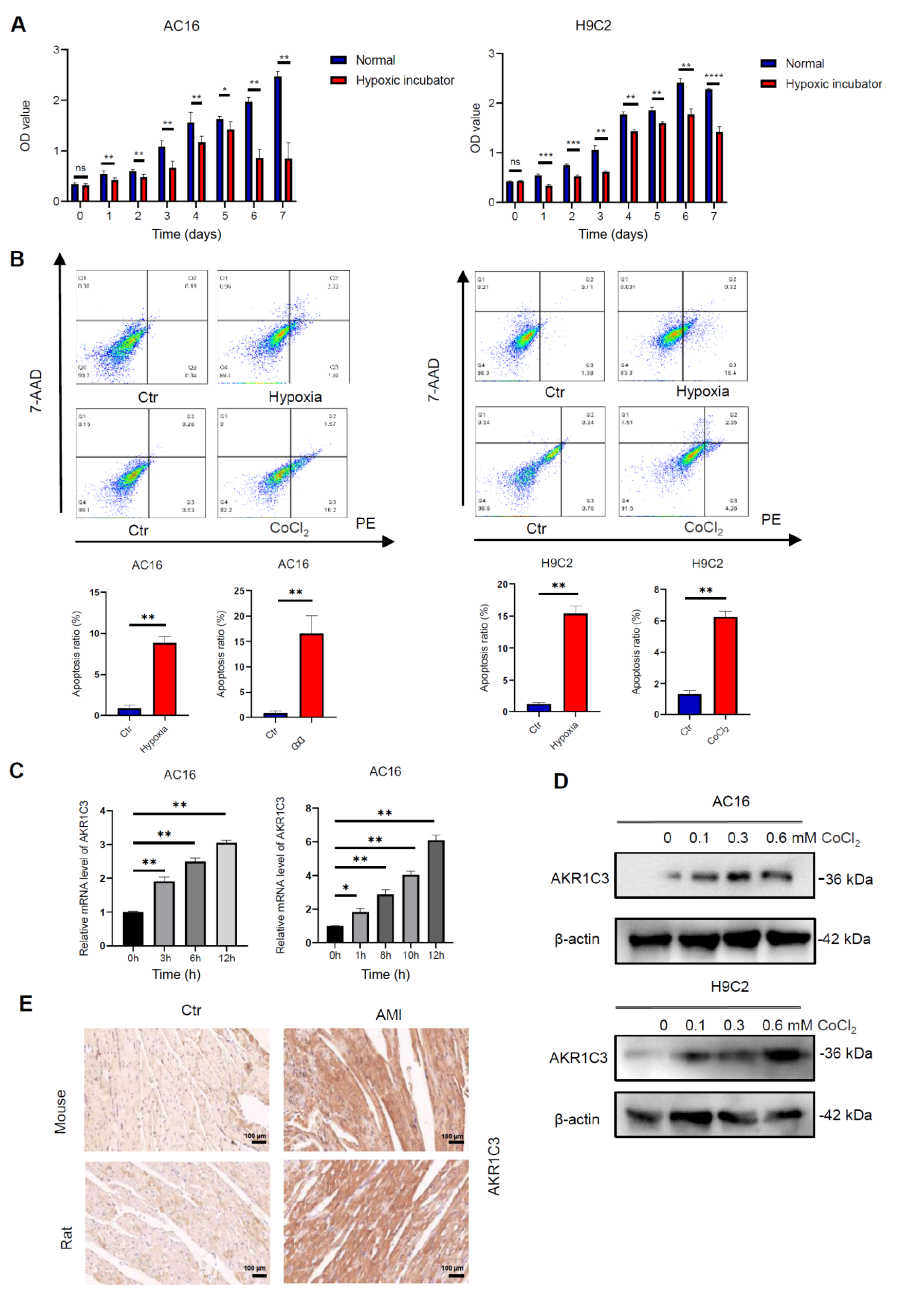
Figure 1 Expression of AKR1C3 in hypoxia-treated cardiomyocytes and myocardial tissue derived from rats and mice with AMI (A) Cell viability was measured by a CCK-8 cell viability assay after 12 h of hypoxia treatment. (B) AC16 and H9C2 cells were either cultured in a hypoxic incubator or treated with CoCl2 (600 μM) for 12 h. Cell apoptosis was detected by flow cytometry. (C) AKR1C3 expression in AC16 cells treated with CoCl2 (600 μM) or subjected to hypoxia for different durations was detected by qRT-PCR. (D) The expression of AKR1C3 in AC16 and H9C2 cells was detected by western blot analysis after treatment with different concentrations of CoCl2. (E) Immunohistochemical analysis of AKR1C3 in the control and AMI groups. Scale bar = 100 μm. Data are presented as the mean ± SD of three independent experiments. *P < 0.05, **P < 0.01.

To confirm the effects of hypoxia on cardiomyocytes, H9C2 and AC16 cells were treated with CoCl _2_ (600 μM) or cultured in a hypoxic incubator (1% O _2_) to mimic a hypoxic microenvironment. Our results revealed that the hypoxic microenvironment inhibited cardiomyocyte viability and induced cardiomyocyte apoptosis in H9C2 and AC16 cells ( Figure 1A,B). We found that the expression of AKR1C3 was upregulated in AC16 and H9C2 cells exposed to a hypoxic microenvironment ( Figure 1C,D). To further validate the role of AKR1C3 in hypoxia-induced cardiomyocyte apoptosis, the expression of AKR1C3 was also detected in cardiac tissues from AMI mice and rats [3]. The IHC results confirmed that the expression of AKR1C3 was upregulated in AMI mouse and rat cardiac tissue ( Figure 1E). These findings indicate that AKR1C3 plays an important role in hypoxia-induced cardiomyocyte apoptosis.

### AKR1C3 increases cardiomyocyte proliferation and cell viability

**Figure 2:**
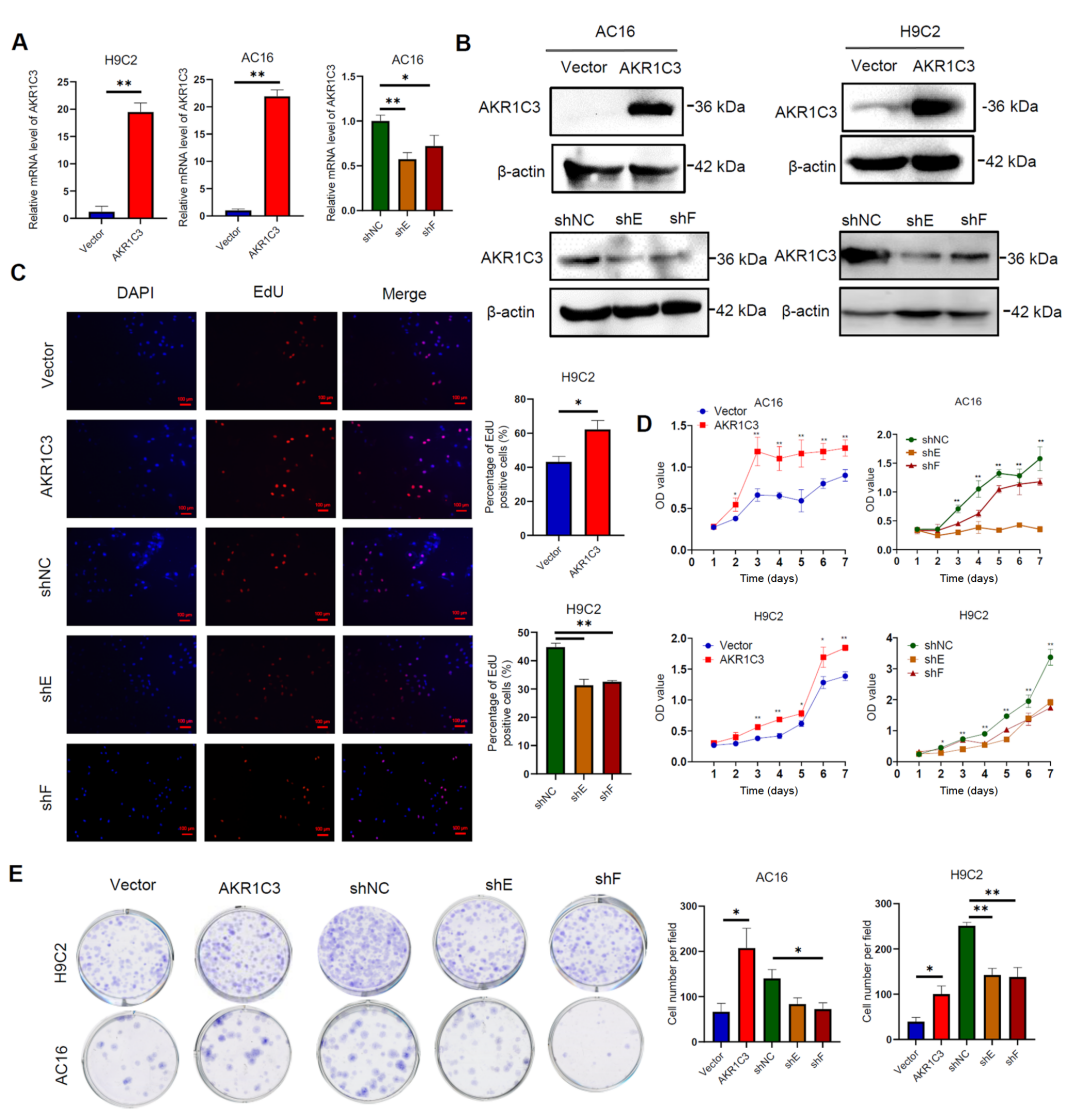
Figure 2 AKR1C3 increases cardiomyocyte proliferation and cell viability (A) The levels of AKR1C3 were detected by qRT-PCR in H9C2 and AC16 cells overexpressing AKR1C3 or AKR1C3 shRNA. (B) The expression of AKR1C3 in AC16 cells expressing AKR1C3 or AKR1C3 shRNA was detected by western blot analysis. (C) The effects of AKR1C3 overexpression and knockdown on cell proliferation were assessed via EdU assay (C), CCK8 assay (D) and colony formation assay (E) in AC16 and H9C2 cells. Scale bar = 100 μm. Data are presented as the mean ± SD of three independent experiments. *P < 0.05, **P < 0.01.

Next, to verify the role of AKR1C3 in cardiomyocytes, H9C2 and AC16 cells were selected for loss- or gain-of-function studies. The efficiency of AKR1C3 overexpression and knockdown was verified via qRT-PCR and western blot analysis ( Figure 2A,B). Our results revealed that the overexpression of AKR1C3 increased cardiomyocyte proliferation, cell viability and colony formation ability ( Figure 2C–E). Conversely, *AKR1C3* knockdown decreased cardiomyocyte proliferation, cell viability and colony formation ability ( Figure 2C–E). These results suggest that AKR1C3 promotes cardiomyocyte proliferation and improves cell viability.

### AKR1C3 protects cardiomyocytes from hypoxia-induced apoptosis and reduces ROS levels and mitochondrial damage

**Figure 3:**
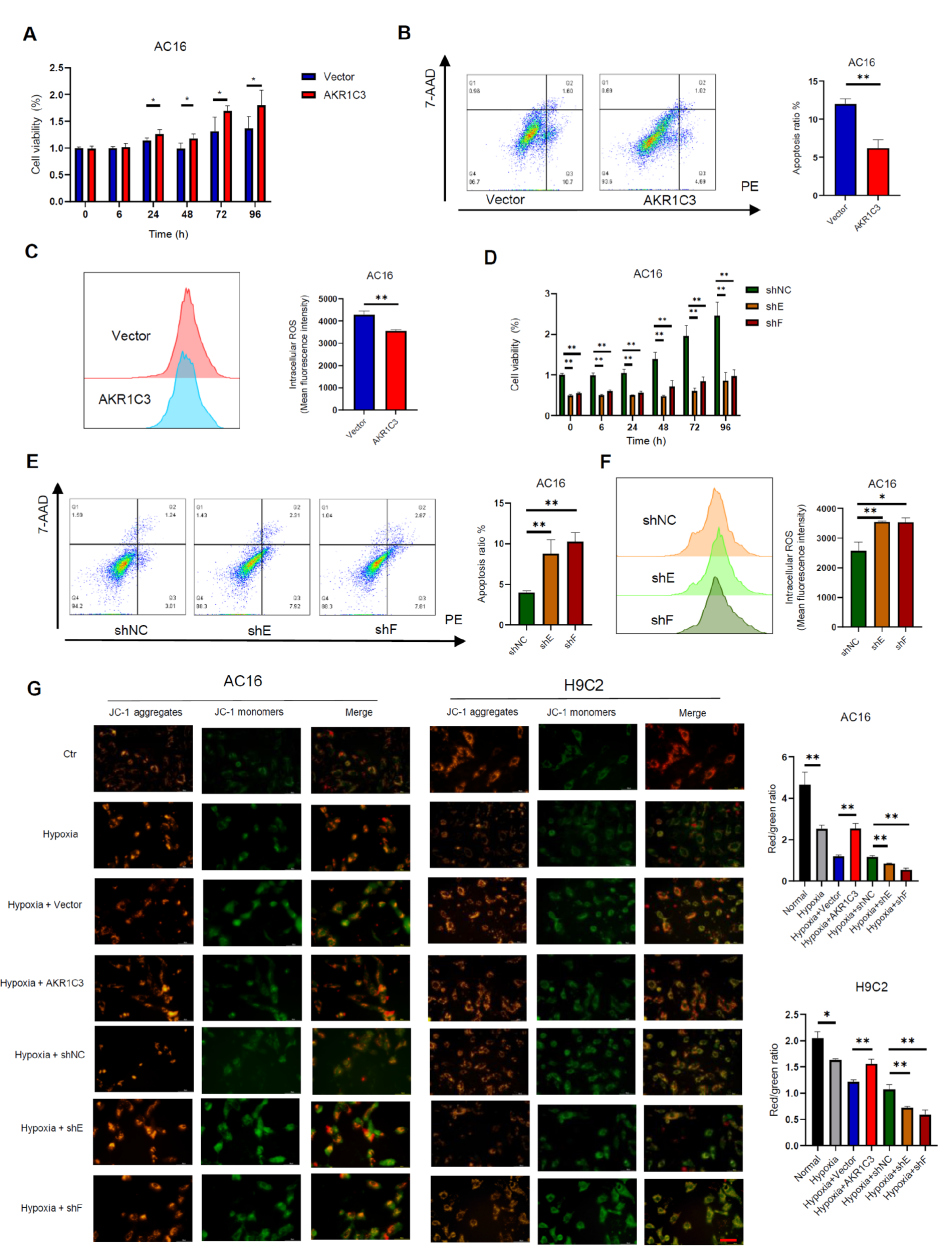
Figure 3 AKR1C3 protects against hypoxia-induced cell apoptosis (A–C) AKR1C3-overexpressing AC16 cells were treated with CoCl2 (600 μM) for 12 h. Cell viability was determined by CCK-8 assay (A). Apoptosis was analyzed by flow cytometry (B). ROS levels were assessed via DCFH-DA staining in AC16 cells via flow cytometry (C). (D–F) AKR1C3-knockdown AC16 cells were treated with CoCl2 (600 μM) for 12 h. Cell viability was determined by a CCK-8 assay (D). Apoptosis was analyzed by flow cytometry (E). The ROS level in AC16 cells was assessed via DCFH-DA staining via flow cytometry (F). (G) The mitochondrial membrane potential was measured by confocal microscopy after the cells were stained with the fluorescent mitochondrial probe JC-1. Scale bar = 50 μm. Data are presented as the mean ± SD of three independent experiments. *P < 0.05, **P < 0.01.

We subsequently evaluated the influence of AKR1C3 on cardiomyocytes in a hypoxic microenvironment. We found that overexpression of AKR1C3 mitigated hypoxia-induced apoptosis and reduced intracellular ROS levels while simultaneously increasing cell viability ( Figure 3A–C). Conversely, *AKR1C3* knockdown markedly exacerbated hypoxia-induced apoptosis, elevated intracellular ROS levels and diminished cell viability ( Figure 3D–F). To further investigate mitochondrial function, we employed the JC-1 fluorescent probe to monitor alterations in the MMP in AC16 and H9C2 cells. The MMP is an indicator of mitochondrial function. Notably, our results indicated that following hypoxia exposure in a hypoxia chamber, overexpression of AKR1C3 partially restored the MMP by shifting JC-1 fluorescence from green monomers to red aggregates, suggesting a protective role of AKR1C3 against hypoxia-induced mitochondrial dysfunction. On the other hand, the JC-1 red/green fluorescence intensity ratio was significantly decreased in both AC16 and H9C2 cells subjected to *AKR1C3* knockdown under hypoxic conditions ( Figure 3G). Collectively, these findings demonstrate that AKR1C3 exerts a protective effect on cardiomyocytes by alleviating hypoxia-induced apoptosis, reducing ROS levels, and preserving mitochondrial integrity.

### ROS inhibitor (MitoQ10) mitigates the increase in mitochondrial superoxide production in cardiomyocytes induced by *AKR1C3* knockdown under hypoxic conditions

**Figure 4:**
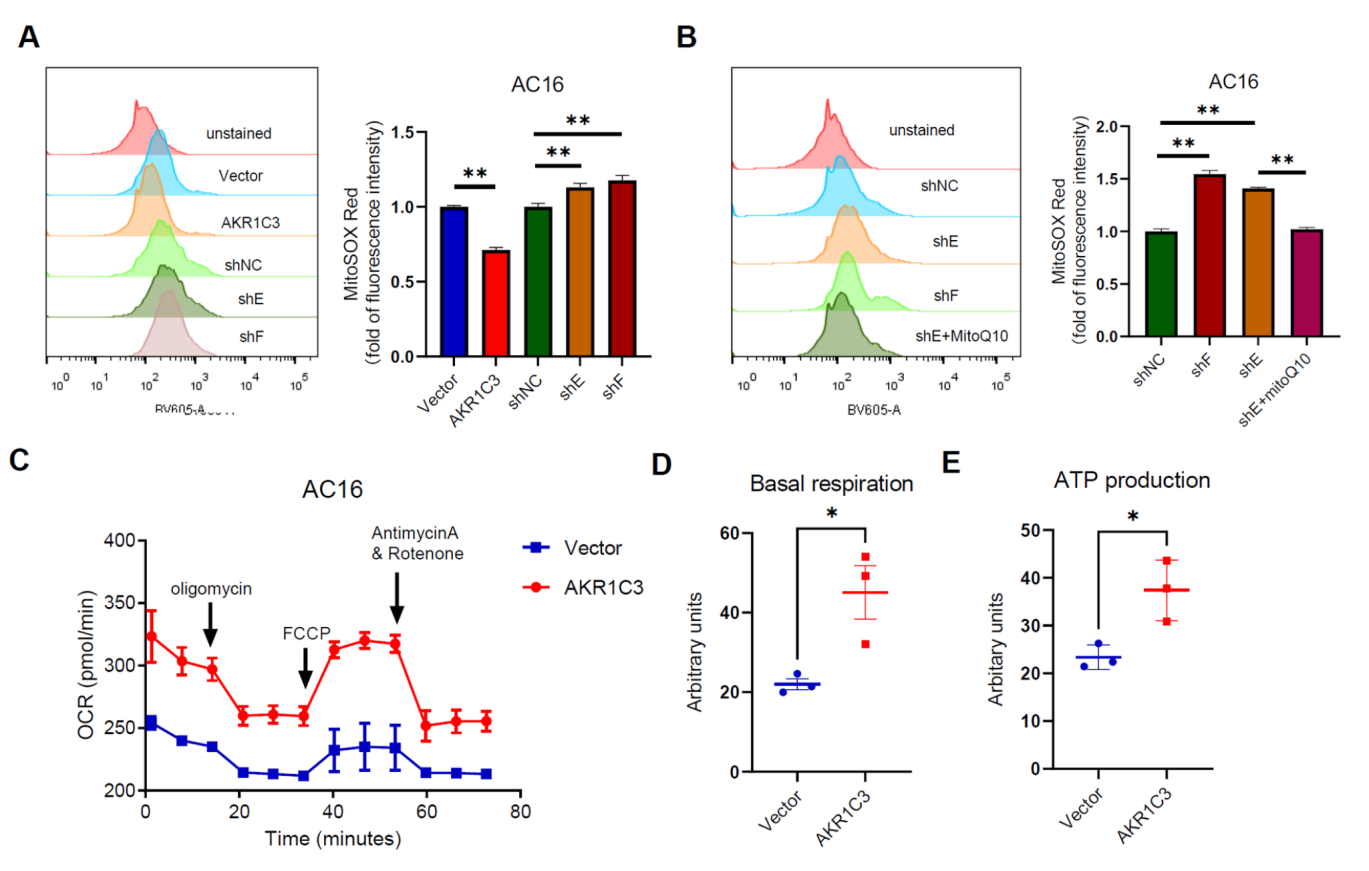
Figure 4 Effect of AKR1C3 on mitochondrial respiration and mitochondrial superoxide production in AC16 cells under hypoxic conditions (A) AKR1C3-overexpressing and AKR1C3-knockdown AC16 cells were exposed to hypoxia for 12 h. The production of mitochondrial superoxide, which was detected with the fluorescent probe MitoSOX Red, was assessed by flow cytometry. (B) AKR1C3-knockdown AC16 cells pretreated with 500 nM mitoQ for 4 h were exposed to hypoxia for 12 h, and mitochondrial superoxide levels were detected by flow cytometry. (C–E) The effect of AKR1C3 overexpression on the OCR was measured with a Seahorse XF24 Analyzer (C). Basal mitochondrial respiration (D) and ATP production (E) were calculated and statistically analyzed (n = 3). *P < 0.05, **P < 0.01.

MitoSOX is a mitochondria-specific indicator of superoxide, and the production of mitochondrial superoxide was quantitatively analyzed via flow cytometry. Therefore, MitoSOX Red dye was used to detect the source of ROS via flow cytometry. Overexpression of AKR1C3 significantly reduced the production of mitochondrial superoxide, whereas *AKR1C3* knockdown increased its production ( Figure 4A). Mitoquinone (MitoQ10) is a mitochondrion-targeted antioxidant that reduces the overproduction of ROS in mitochondria. Our results revealed that MitoQ10 alleviated the increased production of mitochondrial superoxide caused by *AKR1C3* knockdown in AC16 cells under hypoxic conditions ( Figure 4B).

The oxygen consumption rate (OCR) can directly reflect mitochondrial function. To further assess mitochondrial function, real-time measurements of cellular metabolism were performed via a Seahorse XF96 analyzer. Compared with those of control cells, the OCR, ATP production, and basal respiration of AC16 cells overexpressing AKR1C3 were significantly greater ( Figure 4C–E). These findings suggest that under hypoxic conditions, AKR1C3 plays an important role in protecting mitochondrial function.

### AKR1C3 increases Nrf-2 expression through the ubiquitin‒proteasome pathway

**Figure 5:**
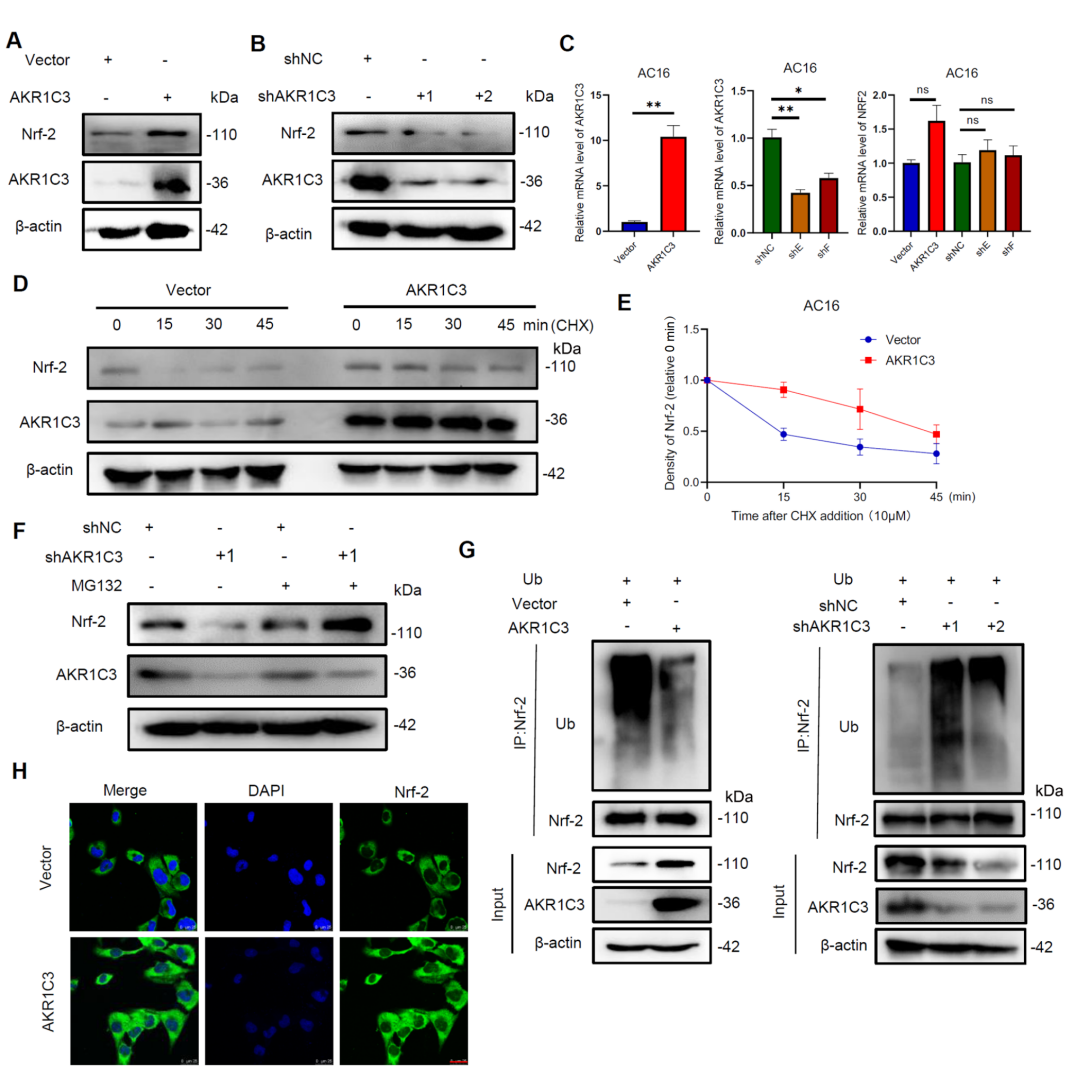
Figure 5 AKR1C3 increases Nrf-2 expression via the ubiquitin-proteasome pathway in AC16 cells (A,B) The expression of Nrf-2 in AKR1C3-overexpressing and AKR1C3-knockdown AC16 cells was detected by western blot analysis. (C) Nrf-2 expression was detected by qRT-PCR in AKR1C3-overexpressing and AKR1C3-knockdown AC16 cells. (D,E) Nrf-2 expression was detected by western blot analysis in AKR1C3-overexpressing AC16 cells treated with cycloheximide (CHX) at the indicated time points. (F) Nrf-2 expression was detected by western blot analysis in AKR1C3-knockdown AC16 cells treated with MG132. (G) Lysates of AKR1C3-overexpressing and AKR1C3-knockdown AC16 cells were immunoprecipitated with an anti-Nrf-2 antibody, and the immunocomplexes were immunoblotted with antibodies against ubiquitinated proteins. (H) Nrf-2 protein expression in AKR1C3-overexpressing AC16 cells was determined by immunofluorescence assays. Scale bar = 25 μm. *P < 0.05, **P < 0.01; ns: not significant.

Nrf-2 is a key factor in cellular oxidative stress, regulating the expressions of antioxidant proteins by interacting with antioxidant response elements (AREs) [20]. Our results revealed that the overexpression of AKR1C3 increased the level of Nrf-2 in hypoxia-treated cardiomyocytes ( Figure 5A), whereas the knockdown of *AKR1C3* had the opposite effect ( Figure 5B). However, AKR1C3 did not affect the expression of *Nrf-2* mRNA in hypoxia-treated AC16 cells ( Figure 5C). To investigate the effect of AKR1C3 on Nrf-2 stability, we treated AC16 cells with the protein synthesis inhibitor cycloheximide (100 μM) and detected Nrf-2 protein levels over time. Our results revealed that the degradation rate of Nrf-2 was lower in the AKR1C3-overexpressing group than in the vector control group ( Figure 5D). AKR1C3 overexpression extended the half-life of the Nrf-2 protein from 15 to 45 min ( Figure 5E). Next, we hypothesized that the ubiquitin–proteasome pathway might be involved in the proteolytic degradation of Nrf-2. After hypoxia-exposed AC16 cells were treated with the reversible proteasome inhibitor MG132 (10 μM), the degradation of Nrf-2 induced by *AKR1C3* knockdown was reversed ( Figure 5F). Additionally, AKR1C3 overexpression led to a reduction in ubiquitinated Nrf-2, whereas *AKR1C3* knockdown resulted in the accumulation of ubiquitinated Nrf-2 ( Figure 5G). Furthermore, overexpression of AKR1C3 increased the expression and nuclear localization of Nrf-2 ( Figure 5H). These results suggest that AKR1C3 promotes Nrf-2 expression by reducing its ubiquitination in hypoxia-treated cardiomyocytes.

### AKR1C3 regulates the NF-κB signaling pathway

**Figure 6:**
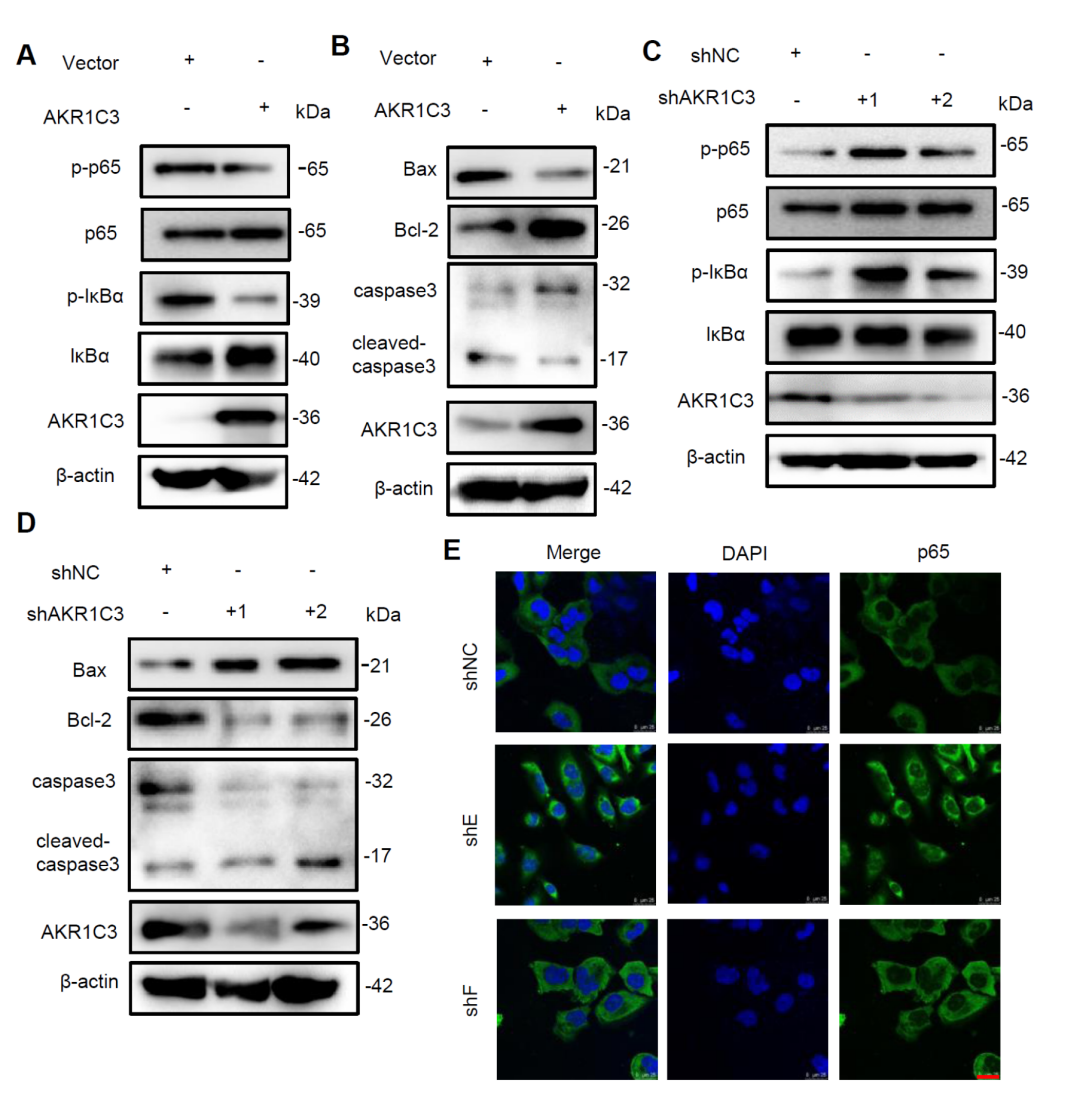
Figure 6 AKR1C3 inhibits NF-κB and Bax/caspase-3 signaling in hypoxia-treated AC16 cells (A–D) The expressions of p-p65, p65, p-IκBα, IκBα, and Nrf-2 in AKR1C3-overexpressing (A) and AKR1C3-knockdown (C) AC16 cells were detected via western blot analysis. The expressions of Bax, Bcl-2, caspase 3, and cleaved caspase 3 in AKR1C3-overexpressing (B) and AKR1C3-knockdown (D) AC16 cells were detected via western blot analysis. (E) p65 localization in the indicated cells was analyzed by immunofluorescent confocal imaging. Scale bar = 25 μm.

NF-κB is a transcription factor that plays an important role in inflammation and apoptosis [ 21, 22]. ROS generation can modulate the NF-κB response [23]. To further explore the mechanisms of AKR1C3 in hypoxic injury, we examined the activation of NF-κB signaling in AC16 cells exposed to hypoxia for 12 h. Western blot analysis revealed that AKR1C3 overexpression prevented the increase in p-p65 and p-IκBα levels induced by hypoxia ( Figure 6A). Conversely, *AKR1C3* knockdown activated the NF-κB signaling pathway in hypoxic injury, as indicated by increased p-p65 and p-IκBα levels ( Figure 6C). Furthermore, knockdown of *AKR1C3* led to increased nuclear translocation of p65 ( Figure 5E). Consistent with previous findings, western blot analysis of apoptosis revealed that in AC16 cells overexpressing AKR1C3, the expressions of proapoptotic proteins (Bax and cleaved-caspase-3) were decreased, whereas the expression of an antiapoptotic protein (Bcl-2) was increased ( Figure 6B). In contrast, in *AKR1C3*-knockdown AC16 cells, the levels of proapoptotic proteins were increased, and the levels of antiapoptotic proteins were decreased ( Figure 6D).

**Figure 7:**
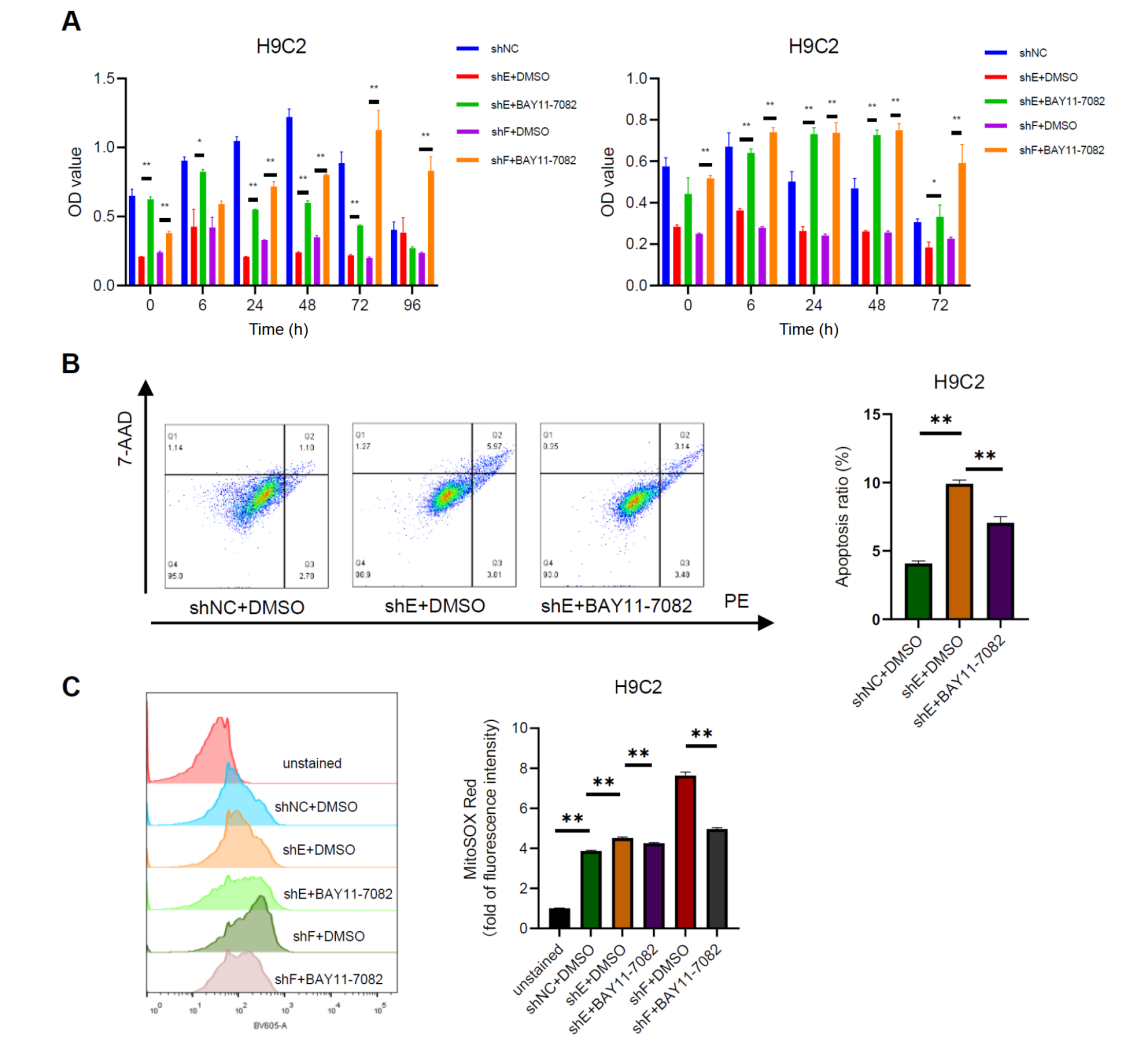
Figure 7 Deactivation of NF-κB signaling attenuates apoptosis, cell viability, and mitochondrial superoxide production induced by *AKR1C3* knockdown in hypoxia-induced H9C2 cells AKR1C3-knockdown H9C2 cells pretreated with or without BAY 11-7082 (5 μM) were exposed to hypoxia or treated with CoCl2 (600 μM) for 12 h. (A) Cell viability was measured by CCK8 assay. (B) Apoptosis was analyzed by flow cytometry. (C) The production of mitochondrial superoxide stained with the fluorescent probe MitoSOX Red was assessed by flow cytometry. **P < 0.01.

To confirm the role of the NF-κB signaling pathway in AKR1C3-mediated cardioprotection in a hypoxic microenvironment, we used BAY11-7082, an inhibitor of NF-κB. Recent studies have shown that BAY 11-7082 can reduce the translocation of p65 in the cell nucleus [24]. Our results revealed that the decrease in cell viability induced by *AKR1C3* knockdown was reversed by treatment with BAY11-7082 ( Figure 7A). In addition, the rates of apoptosis ( Figure 7B) and the production of mitochondrial superoxide ( Figure 7C) induced by hypoxia were reversed by treatment with BAY11-7082. These results suggest that AKR1C3 protects cardiomyocytes from hypoxia-induced apoptosis by regulating the NF-κB signaling pathway.

## Discussion

Hypoxia is a common feature in the pathophysiology of a variety of cardiovascular disorders. For example, heart failure with reduced left ventricular systolic function can cause insufficient oxygen in the body, thereby exacerbating myocardial oxygen deficiency [25]. Atherosclerosis is a chronic inflammatory disease that can increase the risk of myocardial infarction, and the thickness of the arterial wall and plaque inflammation (increasing oxygen demand) induce hypoxia in the intima, consequently diminishing perfusion to the cardiac tissue and further promoting the progression of atherosclerosis [26]. Numerous factors can induce cardiomyocyte apoptosis, including ischaemia‒reperfusion injury, traumatic injury, chemical drugs and radiation therapy [ 27– 29]. Cardiomyocyte apoptosis contributes to the development and progression of heart failure. In this study, we found that the expression of AKR1C3 was upregulated during hypoxia-induced cardiomyocyte apoptosis. Furthermore, we found that AKR1C3 increased Nrf-2 expression via the ubiquitin-proteasome pathway in cardiomyocytes and subsequently inhibited the NF-κB signaling pathway, thereby protecting cardiomyocytes from hypoxia-induced apoptosis and injury.

Oxidative stress is involved in the development and progression of heart failure. An overabundance of ROS can lead to the development and progression of myocardial remodeling and heart failure [ 30, 31]. In this study, we found that hypoxia-induced oxidative stress promoted the release of ROS and cellular injury. ROS overload triggers the opening of the mitochondrial permeability transition pore (mPTP), leading to the loss of the mitochondrial membrane potential and triggering the apoptosis pathway [32]. These effects lead to a decrease in ATP production and OCR. Our results also revealed that hypoxia induced mitochondrial membrane potential dysfunction and Bcl-2/Bax/caspase3 pathway activity. AKR1C3, a well-known member of the aldo-keto reductase (AKR) superfamily, has been studied extensively in cancer-related biological processes, including cell proliferation and chemosensitivity [33]. In nonneoplastic diseases, increasing the expression of AKR1C3 in the kidneys of rats with preeclampsia could reduce the production of ROS in renal tissue, thereby exerting a therapeutic effect on preeclampsia [34]. Although Liang *et al*. [35] reported that AKR1C3 was significantly downregulated in the peripheral blood samples of patients with AMI, 16 samples from the peripheral blood were very small and did not reflect myocardial expression. Therefore, we detected the expression of AKR1C3 in hypoxia-induced cardiomyocytes and myocardial tissues from AMI mice. We found that the expression of AKR1C3 was increased in hypoxia-induced cardiomyocytes and myocardial tissues from AMI mice. Furthermore, we found that AKR1C3 prevented a decrease in ATP production and the OCR in hypoxia-induced cardiomyocytes. These results suggest that AKR1C3 protects cardiomyocytes against hypoxia-induced cardiotoxicity damage by suppressing excessive ROS.

Nrf-2 plays a protective role against many pathological conditions closely related to oxidative stress [36]. In this study, we found that AKR1C3 protects cardiomyocytes from hypoxia-induced apoptosis by regulating the Nrf-2 signaling pathway. We found that overexpression of AKR1C3 increased the stability of the Nrf-2 protein and subsequently suppressed NRF-2 ubiquitination in cardiomyocytes. Under normoxic conditions, Nrf2 is bound by Kelch-like ECH-associated protein 1 (Keap1) and is inactivated through ubiquitination and degradation in the proteasome [37]. In response to oxidative stress, Nrf2 is released from Keap1 binding, translocates to the nucleus and activates a range of cell-protective genes. We speculate that AKR1C3 can stabilize Nrf-2 through Keap1-mediated ubiquitin‒proteasome degradation. In addition, a previous study showed that NRF2/MAFG was able to bind directly to the *AKR1C3* promoter to activate its transcription in HCC cells [38]. Therefore, we speculate that AKR1C3/Nrf-2 can form a positive regulatory loop in cardiomyocytes.

Oxidative stress is considered a trigger of inflammation, one of the most prevalent risk factors for AMI [39]. The Nrf-2 and NF-κB pathways regulate the homeostasis of the cellular redox status. Crosstalk between Nrf-2 and NF-ĸB under stress is a complex phenomenon. Nrf-2 can directly interact with components of the NF-κB signaling pathway, such as by inhibiting the activity of IKK (IκB kinase), thereby preventing NF-κB from translocating from the cytoplasm to the nucleus, which in turn inhibits the transcriptional activity of NF-κB.

Deletion of Nrf-2 is associated with enhanced inflammation transcriptionally regulated by NF-ĸB [40]. NRF-2 deficiency in mouse onic fibroblasts led to the activation of NF-κB [41]. Furthermore, a previous study showed that Nrf-2 inhibits NF-ĸB as a part of a regulatory feedback loop [42]. NF-κB is a pivotal modulator of the inflammatory response [43]. Onai *et al*. [44] reported that inhibition of the nuclear translocation of NF-κB via blockade of IκBα phosphorylation attenuated myocardial ischemia/reperfusion (I/R) injury. In this study, knockdown of *AKR1C3* promoted the phosphorylation of IκBα, which resulted in the release of the NF-κB subunit p65 and subsequent p65 nuclear translocation. *AKR1C3* knockdown-induced cardiotoxicity damage was reversed by treatment with the NF-κB inhibitor BAY11-7082 in the hypoxic microenvironment. Previous studies have shown that BAY 11-7082 significantly reduces the infarct size and preserves myocardial function in a rat model of myocardial I/R injury [45]. These results suggest that AKR1C3 protects cardiomyocytes from hypoxia-induced apoptosis by regulating the NF-κB signaling pathway.

**Figure 8:**
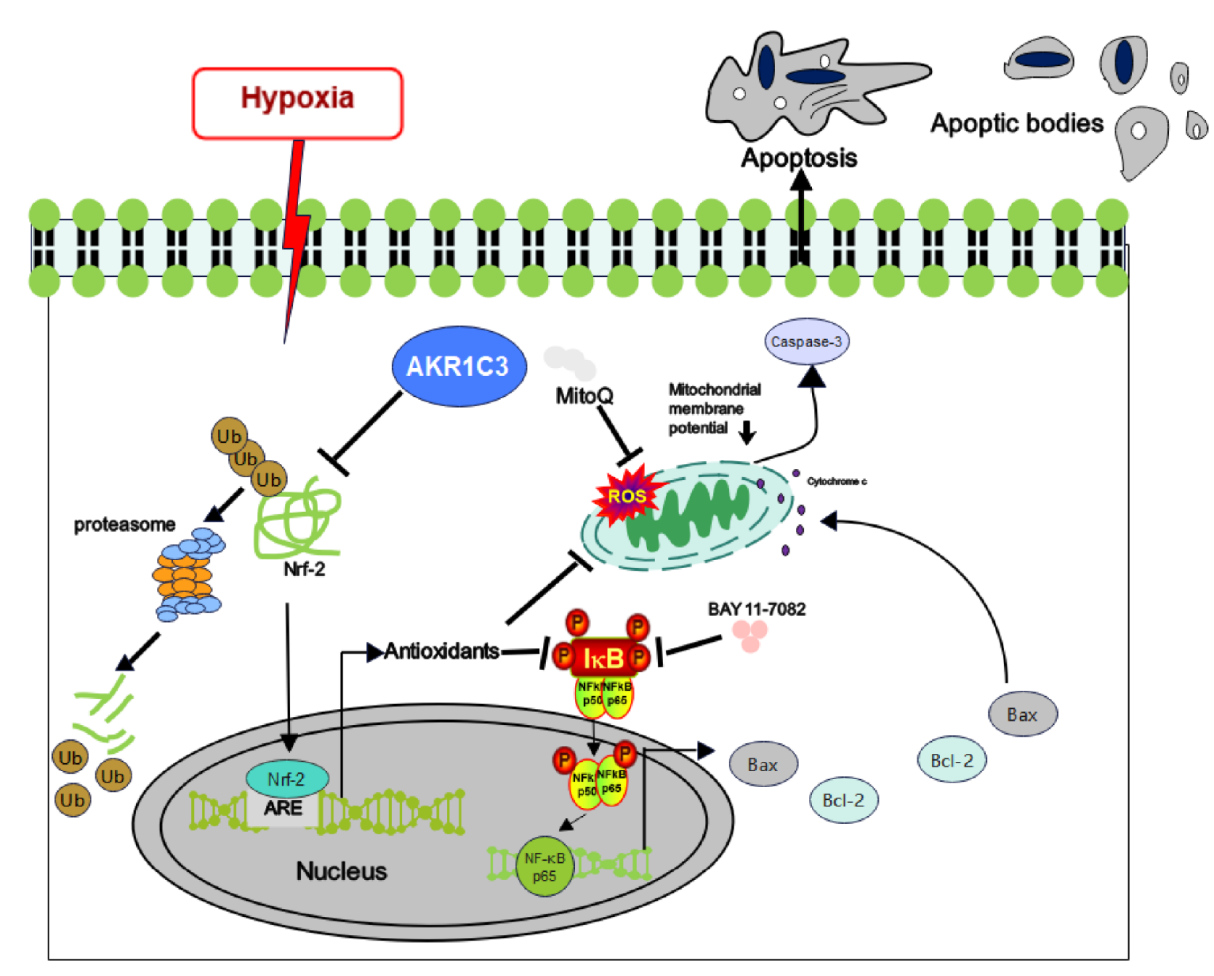
Figure 8 Model of the mechanisms of action of AKR1C3 in hypoxia-induced cardiomyocyte apoptosis AKR1C3 stabilizes Nrf-2, decreases Nrf-2 ubiquitination, influences its degradation, subsequently activates antioxidants and inhibits NF-κB and Bax/caspase-3 signaling.

In conclusion, the expression of AKR1C3 was upregulated in hypoxia-induced cardiomyocytes. AKR1C3 prevents hypoxia-induced cardiomyocyte injury by modulating the NRF-2/NF-κB axis, which provides new insights into the mechanisms underlying myocardial protection ( Figure 8).
